# Ambient air temperature and out‐of‐hospital cardiac arrest: A national case‐crossover study

**DOI:** 10.1111/joim.70144

**Published:** 2026-08-03

**Authors:** Emily Yamron, Marcus Dahlquist, Martin Jonsson, Per Nordberg, Jacob Hollenberg, Leif Svensson, Stefan Agewall, Massimo Stafoggia, Petter Ljungman

**Affiliations:** ^1^ Columbia University Irving Medical Center New York New York USA; ^2^ Institute of Environmental Medicine Karolinska Institutet Stockholm Sweden; ^3^ Department of Cardiology and Clinical Physiology Danderyd Hospital Stockholm Sweden; ^4^ Department of Clinical Science and Education Center for Resuscitation Science Södersjukhuset Karolinska Institutet Stockholm Sweden; ^5^ Department of Physiology and Pharmacology Section for Anaesthesiology and Intensive Care Medicine Karolinska Institutet Stockholm Sweden; ^6^ Department of Medicine Karolinska Institutet Stockholm Sweden; ^7^ Institute of Clinical Sciences, Karolinska Institute of Danderyd Stockholm Sweden; ^8^ Department of Epidemiology of the Lazio Region Health Service Rome Italy

**Keywords:** cardiac arrest, climate change, cold, extreme temperature, heat, out‐of‐hospital cardiac arrest

## Abstract

**Introduction:**

Out‐of‐hospital cardiac arrest (OHCA) is a major health concern. Roughly 300,000 cases occur annually in Europe; overall survival is 11%. Given the disease burden and increasingly extreme temperatures due to climate change, we investigated the association between air temperature and risk of OHCA in Sweden.

**Methods:**

This study employed a time‐stratified case‐crossover design to examine the relationship between daily mean air temperature and OHCA cases from the Swedish Registry for Cardiopulmonary Resuscitation between 2010 and 2019. Using a machine learning model, mean temperatures were estimated on a 1 × 1 km^2^ grid and converted into location‐specific percentiles for Sweden's 290 municipalities. The relationship between temperature and OHCA was estimated using conditional logistic regression.

**Results:**

29,604 OHCA cases (32% women, median age 74 years) were included. The 1st percentile of temperature ranged from −32.7 to −5.98°C; the 99th percentile ranged from 16.6 to 22.8°C. The reference point, where OHCA risk was lowest, was the 86th percentile. The odds ratio (OR) of OHCA comparing 1st percentile of temperature with the 86th was 1.197 (95% confidence interval [CI] 1.066–1.344), and the OR of OHCA comparing the 99th versus 86th percentile was 1.156 (95% CI 1.058–1.263).

**Conclusions:**

Location‐specific extreme ambient air temperatures were associated with greater risk of OHCA in Sweden. This further adds to evidence suggesting that exposure to locally relative temperature extremes may increase risk of OHCA, even when temperatures do not represent extreme temperature events by absolute metrics. These results may be limited by potential discordance between ambient temperature and individual temperature exposure.

## Graphical abstract

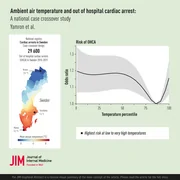

**Abbreviations**
CI: confidence interval
ECG: electrocardiogram
EMS: emergency medical services
MMP: minimum morbidity percentile
OHCA: out‐of‐hospital cardiac arrest
OR: odds ratio
PEA: pulseless electrical activity
PM_2.5_
: fine particulate matter, defined as particles measuring 2.5 µm in diameter or smaller
VT/VF: ventricular tachycardia/ventricular fibrillation

## Introduction

Out‐of‐hospital cardiac arrest (OHCA) is a leading cause of global mortality and the third leading cause of death in Europe [1]. It is estimated that 300,000 people suffer OHCA in Europe annually; the incidence varies by country between 67 and 170 per 100,000 person‐years [1, 2]. Given the significant burden of disease posed by OHCA, there is a growing interest in how environmental factors, such as air pollution and ambient temperature, may increase the risk of OHCA [3, 4]. Climate change notably affects ambient temperature, increasing the risk of extreme heat as well as higher variability in temperature [5, 6]. From previous research, both cold and hot temperatures have been associated with health outcomes in a U‐shaped relationship [7]. Currently, studies investigating ambient temperature and OHCA are relatively few, and a nationwide approach has only been taken in three countries: Korea, Israel, and Japan [8, 9, 10, 11]. Thus, the robustness of the relationship between ambient air temperature and OHCA is incompletely understood. In particular, research on this topic is lacking in colder continental climates such as those covering much of Northern Europe and North America. Meanwhile, extreme temperature events, particularly extreme heat events, are growing more common and are accentuated near the poles [5]. Using high‐quality national registers, this research aims to better understand the relationship between temperature and OHCA in Sweden.

## Methods

### Study population

We collected OHCAs across Sweden occurring between 2010 and 2019 from the Swedish Registry for Cardiopulmonary Resuscitation. This registry has been described in detail previously [12, 13, 14]. Briefly, it is a national registry of all cases of OHCA where emergency medical services (EMS) or a bystander initiated cardiopulmonary resuscitation. EMS centers for all of Sweden report data using a standardized form, including the time, date, and location of the arrest, age, sex, first electrocardiogram (ECG) rhythm, and whether OHCAs were medical or nonmedical (e.g., trauma, intoxication, asphyxiation, and drowning). Data on whether the presenting rhythm was shockable (i.e., ventricular tachycardia/ventricular fibrillation, VT/VF) or non‐shockable (asystole or pulseless electrical activity, PEA) are also included. Inclusion criteria for this study were all cases of medical OHCA in adults (age ≥18 years) registered in the Swedish Registry for Cardiopulmonary Resuscitation during our study period. Exclusion criteria were cases without a location or date associated with the cardiac arrest. The address where EMS picked up the patient was used as the location for the exposure assessment.

### Exposure assessment

We used a national machine learning model to estimate daily mean outdoor air temperature and fine particulate matter air pollution (PM_2.5_). The model, described in detail elsewhere, utilizes machine learning to predict daily air pollution and temperature levels based on satellite data (including aerosol optical depth, land surface temperature, greenness, and light at night), land use, air pollution sources, population data, and atmospheric models and is cross‐validated against real temperature and air pollution (*R* ^2^ = 0.69) data [15]. These predictions are made on a 1 × 1 km^2^ grid. The pick‐up address for each case was geocoded, and exposures for the days of case and control periods (defined later) were estimated for the 1 × 1 km^2^ cell of the geocoded pick‐up address. Taking into account the considerable variability in temperatures at different latitudes in Sweden and existing research that suggests adaptation to local climate, daily temperature exposure was characterized based on municipality‐specific percentiles. Percentiles were drawn from the annual distribution of temperatures in each municipality. Air pollution was quantified as the absolute PM_2.5_ value in µg/m^3^ [16, 17, 18, 19].

### Case‐crossover design

We used a time‐stratified case‐crossover design to investigate the relationship between temperature and OHCA. For each patient, we compared temperature exposure during case and control periods using the percentile of daily mean temperature for the same individual in the same location. For each individual, the case period corresponded to the day when the OHCA occurred; control periods were selected to be the same day of the week within the calendar month of the case, both before and after the case occurred. For example, a case occurring on a Monday in January 2015 would have as control days all other Mondays in January 2015. This strategy accounts for confounding effects that could occur by day of the week, long‐term time trends, and seasonal patterns [20, 21]. Furthermore, as each case is compared with itself on different days, all individual‐level time‐invariant (or slowly varying) characteristics (e.g., age, sex, lifestyles, comorbidities, and socio‐economic status) are automatically adjusted for.

### Statistical analysis

We modeled the association between daily mean temperature percentile and OHCA with conditional logistic regression. The model compared the temperature percentiles for the specific case and control days for each individual. We first modeled the nonlinear association between exposure and outcome using a natural cubic spline with knots at the 10th, 75th, and 90th percentiles of temperature. We then calculated the odds ratio (OR) and 95% confidence interval (CI) of OHCA for 99th percentile versus the minimum morbidity percentile (MMP, the temperature percentile where the OR of OHCA is at its nadir) to evaluate the relationship between heat and OHCA, and 1st percentile versus MMP to evaluate the relationship between cold and OHCA. We modeled exposure for same‐day temperature (Lag 0) and previous week's temperature (Lags 0–6) to capture associations with very short exposure periods as well as longer induction periods. We did not extend the lag analysis beyond 6 days so as not to interfere with the time‐stratified strategy for control day selection.

### Sensitivity analysis

We conducted several sensitivity analyses. To explore the robustness and relevance of the specified exposure period in relation to OHCA, we evaluated different lag patterns, including lags of 0–1 day and 0–2 days. To assess the sensitivity of our results to modeling the nonlinear response to temperature, we also evaluated the temperature effect using differing knot placements and numbers compared to our main model knot placement of the 10th, 75th, and 90th percentiles. Alternate models were run using knots placed at the 10th, 50th, and 90th percentiles; 25th, 50th, and 75th percentiles; and 25th, 50th, and 90th percentiles. In addition, we ran one model with four knots at the 10th, 50th, 75th, and 90th percentiles, as well as one model with five knots at the 10th, 25th, 50th, 75th, and 90th percentiles. To account for possible confounding by air pollution, we ran an analysis adjusting for PM_2.5_ air pollution. By examining the association between temperature and OHCA using only cases where EMS pickup occurred at a person's home, we were more likely to reduce our misclassification exposure during case and control periods as the likelihood of being at the same location (home) during the three to four control periods was assumed to be higher. To capture more extreme temperatures, we conducted an additional sensitivity analysis dividing the year into warm season (April–September) and a cold season (October–March), and temperature exposure was defined based on season‐ and municipality‐specific percentiles.

Finally, we conducted a year‐by‐year analysis to assess whether the growing presence of extreme temperatures over time impacted the relationship between temperature and OHCA.

### Effect modification

We stratified our analysis by different effect modifiers that may be related to the association between ambient temperature and OHCA. These included age (above the median vs. median and younger), sex, urban, or rural location of pickup (based on municipality), first ECG rhythm, and region (North, Central, and South) to examine whether the association between temperature and OHCA was modified by these variables. At the 1st percentile of temperature, effect modification was evaluated with a lag of 0–6 days; at the 99th percentile, effect modification was evaluated with a lag of 0 days. The difference in beta values between strata was computed to assess whether these factors modified the association between temperature and OHCA.

## Results

### Demographics

The registry included 43,928 cases of cardiac arrest; 13,626 were excluded for a nonmedical etiology, and an additional 698 were excluded as they occurred in subjects <18 years of age. A total of 29,604 cases of OHCA reported in the registry were eligible for inclusion during 2010–2019. Of these, 15,669 occurred in the cold season (October–March) and 13,935 occurred in the warm season (April–September). A majority of cases occurred in the South (*n* = 15,512, 52.4%), compared with the Central (*n* = 11,313, 38.2%) and North (*n* = 2779, 9.4%). The median age was 74 (IQR 65–83), and 68% were male. Full demographic information can be found in Table 1.

**Table 1:** Demographic and clinical characteristics.

Characteristics	*N* = 29,604^a^
Age	
Median (Q1, Q3)	74 (65, 83)
Sex	
Man	20,019 (68%)
Woman	9583 (32%)
Missing	2
First ECG	
VT/VF	7729 (27%)
Asystole/PEA	21,437 (73%)
Missing	438
Witnessed arrest	20,133 (69%)
Missing	368
Location of OHCA	
Home	21,322 (72%)
Public place	4886 (17%)
Other location	3387 (11%)
Missing	9

*Note*: Q1: first quartile, Q3: third quartile.

Abbreviations: ECG, electrocardiogram; OHCA, out‐of‐hospital cardiac arrest; PEA, pulseless electrical activity; VT/VF, ventricular tachycardia/ventricular fibrillation.

^a^ *n* (%).

### Exposure

Year‐round, the 1st percentile of mean daily temperature ranged from −32.7°C in Pajala to −5.98°C in Gotland. The 50th percentile of mean daily temperature ranged from −0.6°C in Kiruna to 8.8°C in Malmö. The minimum morbidity temperature for same‐day exposure (lag = 0), that is, where the risk of OHCA was lowest, was found to be the 86th percentile. The 86th percentile corresponded to temperatures ranging from 10.2°C in Arjeplog to 17.4°C in Malmö. The 99th percentile ranged from 18.46°C in Kiruna to 24.31°C in Sundbyberg. The median fine particulate matter (PM_2.5_) was 7.25 µg/m^3^ (IQR 5.61–9.34 µg/m^3^).

### Association between temperature and OHCA

**Fig. 1:**
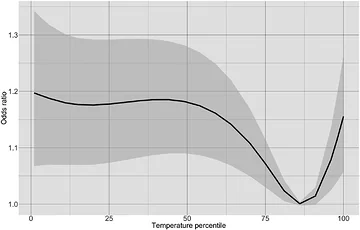
Exposure response function for same‐day temperature percentile and OHCA. This figure shows the OR and 95% confidence interval produced by conditional logistic regression modeling using a lag = 0 days. Broadly, the association between temperature and OHCA follows a U‐shape, with the strongest associations occurring at temperature extremes.

**Fig. 2:**
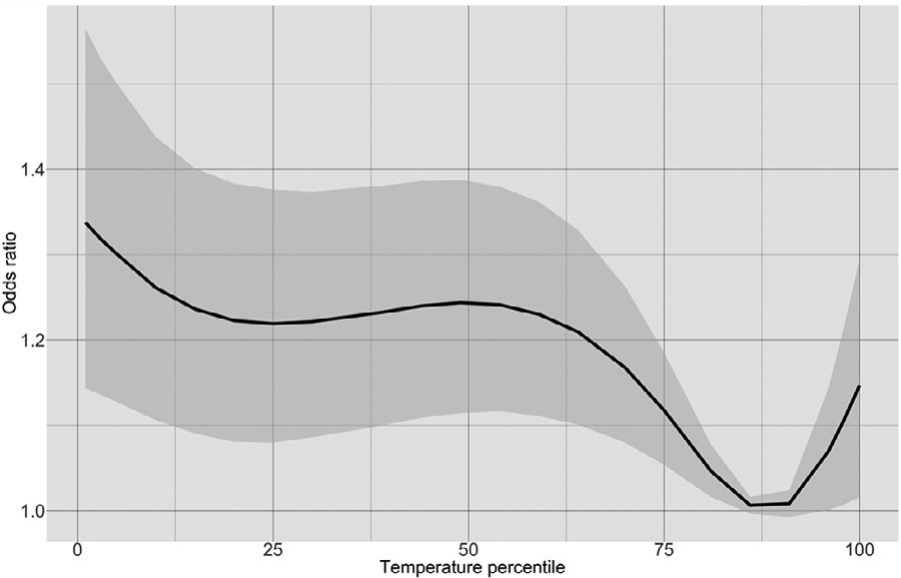
Exposure response function for cumulative temperature exposure lag of 0–6 days and OHCA. This figure shows the OR and 95% confidence interval produced by conditional logistic regression modeling using a cumulative lag = 0–6 days, meaning that it accounts for temperatures in the week leading up to the case or control day. The association between extreme cold and OHCA is slightly more pronounced than in the same‐day model, but otherwise the exposure response functions are similar.

We observed an association between same‐day cold exposure and higher risk of OHCA when comparing the 1st percentile to the MMP (OR = 1.19, 95% CI = 1.07–1.34). An association between same‐day heat exposure and higher risk of OHCA, comparing the 99th percentile to MMP, was also observed (OR = 1.16, 95% CI = 1.06–1.26). We observed the strongest association between cold and OHCA when using a lag of 0–6 days (OR = 1.34, 95% CI = 1.14–1.56); the strongest association between heat and OHCA was observed with same‐day temperature exposure. The exposure response curve showing the relationship between same‐day temperature percentile and OHCA can be seen in Fig. 1, whereas the exposure response curve showing the relationship between cumulative temperature exposure over a lag of 0–6 days and OHCA can be seen in Fig. 2.

Analysis restricted to cases occurring at home showed a similar association between temperature and OHCA to our main model, as did one restricted to only cases occurring away from the home (extended data Fig. 1a,b). Adjusting for PM_2.5_ air pollution resulted in, overall, a similar exposure response curve (extended data Fig. 1c). The exposure response functions using four or five knots were consistent with our main finding (which only used three knots located at 10th, 75th, and 90th percentiles). When using three knots located at different percentiles (10th, 50th, and 90th; 25th, 50th, and 75th; and 25th, 50th, and 90th), we were unable to capture the association between heat and OHCA (extended data Fig. 1d–f). Restricting analysis to the warm season, we found similar associations between same‐day temperature percentile and OHCA at both extremely hot temperatures and extremely cold temperatures (extended data Fig. 2a,b). In restricting analysis to the cold season, we were unable to capture the association between cold temperatures and OHCA. Examining a lag of 6 days during the cold season, we observed a trend toward an association between extreme cold and OHCA, but it did not reach statistical significance (extended data Fig. 2c,d). There were no observed year‐on‐year changes of the odds of OHCA at the 1st or 99th percentile versus MMP (extended data Fig. 3a,b).

### Effect modification

**Fig. 3:**
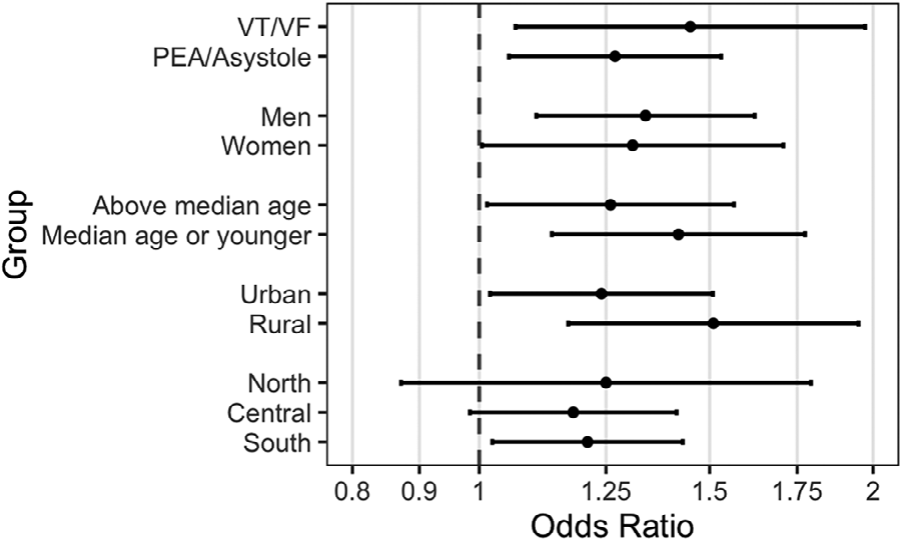
Effect modification by select variables: 1st percentile versus MMP, cumulative lag = 0–6 days. This figure depicts the OR and 95% confidence interval (CI) comparing OHCA at the 1st percentile to the MMP over a cumulative lag of 0–6 days among select subgroups. Lag of 0–6 days was used in the cold due to the slightly stronger association compared to same‐day temperature. There are no significant differences in the relationship between extreme cold and OHCA by gender, age, primary rhythm, urbanization, or region. The odds ratio is plotted on the log scale. PEA, pulseless electrical activity; VT/VF, ventricular tachycardia/ventricular fibrillation.

**Fig. 4:**
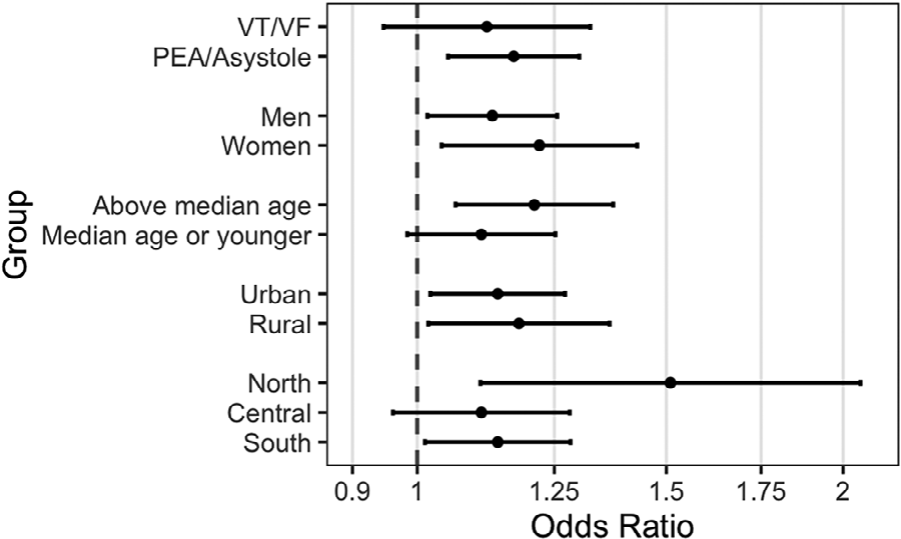
Effect modification by select variables: 99th percentile versus MMP, lag = 0 days. This figure depicts the OR and 95% confidence interval (CI) comparing OHCA at the same‐day 99th percentile to the MMP. There are no significant differences in the relationship between extreme heat and OHCA by gender, age, primary rhythm, or urbanization. The relationship between extreme heat and OHCA trended toward a significant difference when examining effect modification by region. The odds ratio is plotted on the log scale. PEA, pulseless electrical activity; VT/VF, ventricular tachycardia/ventricular fibrillation.

In both extreme cold and extreme heat, no significant effect modification by gender, age (above/below median), primary rhythm, or urbanization of pickup location was seen (all *p* > 0.05, Figs. 3 and 4). In extreme heat, the effect of temperature on OHCA had a near significant effect modification by region, with the North having higher odds of OHCA than the Central region (*p* = 0.069) or South region (*p* = 0.083).

## Discussion

In this study of 29,604 OHCAs across Sweden, associations between location‐specific extreme heat and extreme cold and OHCA were observed. Broadly, both increases and decreases from an optimum ambient air temperature were associated with greater risk of OHCA. More specifically, the exposure response curve demonstrated three distinct patterns. At temperatures roughly below the 50th location‐specific percentile, risk of OHCA was elevated compared to the MMP; temperature percentiles between the 1st and 50th percentiles were associated with roughly equal risks. At temperatures above the MMP, the risk of OHCA rose sharply. The association between cold temperatures and OHCA was strongest with a lag of 0–6 days, whereas the association between hot temperatures and OHCA was strongest with a lag of 0 days (i.e., same‐day exposure).

Temperature exposure and risk of OHCA was not affected by adjustment for PM_2.5_ air pollution, and we did not observe that gender, age, primary rhythm, or urbanization significantly modified the effect of the relationship between heat or cold and OHCA. There was a trend toward significant effect modification by region in the heat, with a higher OR of OHCA at the 99th percentile observed in the North than in the Central or South of Sweden. Additionally, no year‐on‐year change in the relationship between extreme temperature and OHCA was observed.

This study adds to existing research suggesting relative temperature extremes are associated with OHCA. In a recent systematic review of environmental exposures and sudden cardiac death, 11 studies found associations between both extreme cold and extreme heat and OHCA [3, 10, 11, 22, 23, 24, 25, 26, 27, 28, 29, 30]. However, the authors note that among existing studies, the association between cold and OHCA is typically larger than the association between heat and OHCA. In an extreme case, one study that examined OHCA in Japan, the relative risk of OHCA comparing the 1st percentile and the MMP of the 84th percentile was 2.10 (95% CI = 1.84–2.40) and 99th versus MMP was 1.06 (95% CI = 1.01–1.12) [11]. By contrast, we observed only a slightly stronger association between extreme cold and OHCA than heat and OHCA in our main model. This is similar to findings in other recent studies [23, 25, 29, 31]. For example, in research examining OHCA in Tokyo, Osaka, and Fukuoka, Japan, the RR of OHCA at the 95th percentile compared to the reference range of 25th–75th percentiles was 1.11 (95% CI = 1.04–1.18), whereas the RR of OHCA at the 5th percentile versus the 25th–75th was 1.20 (95% CI = 1.16–1.25) [31]. Additionally, in several of the remaining studies, the heat effect was modestly larger than the cold effect on OHCA [24, 27, 28]. The cold effect we observed was more pronounced when examining longer lags, which is consistent with prior studies [23]. Although some research suggests a synergistic effect between temperature and air pollution, and previous research has demonstrated the association between air pollution and OHCA [14, 32], we observed a similar risk of OHCA in extreme heat when observing the simultaneous effect of temperature and PM_2.5_ air pollution. In a previous study of air pollution and OHCA in this population, PM_2.5_ was positively associated with risk of OHCA but weakly correlated with air temperature [14]. Although it is likely that both air pollution and temperature are associated with OHCA risk, the lack of correlation between the two environmental exposures indicates no mutual confounding in the present dataset. In two studies, age >65 years significantly modified the effect of heat on OHCA [27, 29]. The exposure in these studies was defined as a heatwave lasting at least 2 days in one study and cumulative exposure over the 2 weeks preceding an OHCA in the other. That we do not see such effect modification in our model, which uses same‐day temperature, may suggest that age modifies the effect of temperature on OHCA only during sustained exposure to extreme heat.

Previous research has suggested that risk of myocardial infarctions is elevated in the cold; given that OHCA is strongly associated with ischemic disease, it is possible that OHCA risk in the cold is driven by ischemic disease [25, 26, 33, 34]. In extreme heat, thermal stress leads to increased sweating and dehydration, prompting increased sympathetic activity to maintain cardiac output, which may increase the risk for ischemia [35]. Recent research has demonstrated that greater myocardial blood flow is required for increases in core body temperature associated with ambient heat. In subjects with coronary artery disease, this produced asymptomatic ischemia [36]. Alternative explanations that have been proposed for increased risk of OHCA in extreme heat include electrolyte derangement and inability to maintain cardiac output [35, 37, 38, 39]. Evidence for these mechanisms might be supported by increased OHCA risk in extreme heat among people with renal disease or congestive heart failure.

It is worth noting that temperature extremes are not defined by absolutes but are relative to location, likely due to a combination of factors, including the physiologic strain of change in temperature, as well as societal adaptations that impact the experience of temperature. For example, in warm climates, architecture often fosters natural shading and ventilation; by contrast, infrastructure in cold climates insulates heat, and active cooling mechanisms are sparse [40, 41, 42]. Thus, although 24°C may not be extreme in the tropics, in parts of Sweden, where this represents the 99th percentile of annual temperatures, such weather may be associated with worse outcomes not only due to physiologic strain but also lack of social adaptation. Differences in physiologic and societal adaptation may also underlie the near‐significant differences seen in the relationship between temperature and OHCA when broken down by region, as the North appeared to have higher odds of OHCA at the 99th percentile than the Central or South regions. Although this observation is limited by small sample size in the North, it is hypothesis generating and worthy of further investigation. Meanwhile, extreme temperature events grow longer, hotter, and more extreme due to anthropogenic climate change, heightening the risk of exposure, even in cooler climates [43, 44, 45, 46].

We observed a triphasic pattern of associations with a stable association between cold temperatures at 1st–50th percentile and OHCA, before observing a clear V‐shaped exposure response demonstrating clear increases in risk with colder or hotter temperatures compared to the MMP. The explanation for the observed plateau of increased risk between 1st and 50th percentile compared to MMP is unclear and not immediately discerned by the data. A similar pattern was observed in one study in Taiwan, where there is an apparent plateau at the coldest temperatures (ranging from ∼15 to 17°C), but its significance was not commented on [24]. Speculatively, colder outdoor temperatures might reflect a greater likelihood of remaining indoors on both case and control periods; however, we had no ability to test this hypothesis with our existing data.

There are several limitations to our study that impact our ability to interpret the results. The first is the potential for exposure misclassification, wherein the assigned ambient temperature is not reflective of an individual's actual temperature exposure. Because the temperature percentiles used reflect the outdoor temperature at the site of a subject's cardiac arrest, an individual's actual exposure might deviate either if they spent most of their time in a different location, or if they were indoors. Although ambient temperature does not equal a subject's actual exposure perfectly, such as in circumstances where subjects are indoors, this deviation would likely bias the result toward a null finding. Indoor temperatures can be controlled and therefore fairly constant between case and control days. If exposure were misrepresented such that a true exposure was to a relatively stable indoor temperature, it would be expected that OHCAs would be randomly distributed across case and control days and thus result in null associations. Yet our data clearly indicate an association between temperature and OHCA despite this limitation. It is possible that, in particular at the coldest temperatures, people are spending less time outdoors. This may contribute to the leveling off of the association between temperature and OHCA seen at the lowest temperature percentiles, though risk is still elevated relative to the MMP. However, one of the benefits of the case‐crossover model is that comparisons are being made within a single individual within a relatively time‐restricted period (i.e., all observations are in the same calendar month). Accordingly, behavioral changes such as differential time spent outdoors based on temperature between case and control days are likely sufficiently limited that it is reasonable to make this comparison. Moreover, despite its limitations, ambient air temperature is a well‐recognized environmental exposure studied for outcomes, including all‐cause and cardiac‐specific causes of mortality [7, 47]. Ambient air temperature as an exposure also has several advantages: It is an exposure that can be used to establish policy measures like heat warning systems and is less prone to bias from omitted covariates associated with individual lifestyle or behavioral factors.

We attempted to explore the potential for exposure misclassification due to a subject spending most of their time at a location other than the site of their arrest by using one model examining only cases occurring at home and one using only cases outside the home. Both produced results similar to our main model. Moreover, because our main model utilizes a lag of 0 days, the extent of any possible exposure misclassification is more limited than for longer lags. Additionally, this dataset lacks information about subjects’ cardiovascular risk factors. Because the design of this study uses individuals as their own controls, their risks (including both medical conditions and behaviors such as smoking or exercise) are likely stable within the analytical timeframe of a month. However, the absence of this data means we are unable to comment on which individuals might be at greatest risk for an OHCA during extremely hot or cold days; further research using patient registries may be able to elucidate personal risk factors for OHCA in the setting of extreme temperature. Additionally, understanding comorbidities that confer a higher risk of OHCA in extreme temperatures may be hypothesis generating for underlying pathophysiologic pathways. For example, in the cold, we see that the risk of OHCA is higher among cases with a presenting rhythm of VT/VF. Cardiac OHCAs commonly present with ventricular tachyarrhythmias that progress to asystole [48]. Although a presenting rhythm of asystole or PEA does not exclude a cardiac etiology, presenting rhythms of VT/VF may be concerning for an underlying intrinsic cardiac cause.

The current research also has major strengths. By examining a large national dataset of OHCA and high‐resolution exposure, we were able to study the relationship between temperature exposure and OHCA in a large area with diverse temperature ranges; the high spatial resolution and utilization of same‐day exposure help decrease the risk of exposure misclassification. The use of the Swedish Registry for Cardiopulmonary Resuscitation, a national database that captures the vast majority of OHCAs in the country, provides a comprehensive outcomes dataset with low risk of selection bias. Additionally, by using municipality‐specific percentiles rather than absolute temperature, we are able to assess locally relevant temperature extremes that pose an increased risk of OHCA over a large spatial area. Optimum ambient air temperature has been shown to differ widely, and this strategy allows for identification of locally relevant temperatures that are associated with elevated risk of OHCA. Unlike air pollution, where exposure to increased pollution is hazardous regardless of what baseline exposure is, because there is both individual biological and societal adaptation to temperature, absolute temperature thresholds associated with increased risk differ by location [7]. Using relative percentiles, a strategy commonly employed in multicity and multicountry analyses, allows for the identification of locally relevant temperatures associated with higher and lower morbidity [49].

Overall, this case‐crossover study of OHCAs in Sweden found that risk of OHCA increases with same‐day exposure to both extreme cold and extreme heat. Importantly, OHCA risk in extreme heat was elevated even at temperatures below commonly used heat wave thresholds. Future directions for research include investigating what individual factors, including co‐morbid conditions as well as physiologic and societal adaptations, are associated with an elevated risk of OHCA in extreme temperatures. Additionally, to validate the results of this study, further work should be done to understand the extent to which ambient air temperature effectively approximates individual temperature exposure.

## Author contributions

Emily Yamron was responsible for data analysis and manuscript drafting. Marcus Dahlquist was responsible for project design, data analysis, and manuscript drafting. Martin Jonsson was responsible for project design, acquisition, and editing of the manuscript. Per Nordberg was responsible for project design, data acquisition, and editing of the manuscript. Jacob Hollenberg was responsible for project design, data acquisition, and editing of the manuscript. Leif Svensson was responsible for project design, data acquisition, and editing of the manuscript. Stefan Agewall was responsible for project design, data acquisition, and editing of the manuscript. Massimo Stafoggia was responsible for project design, data acquisition, supervision of the data analysis, and editing of the manuscript. Petter Ljungman was responsible for project design, data acquisition, supervision of the data analysis, and editing of the manuscript.

## Conflict of interest statement

The authors declare no conflicts of interest.

## Funding information

E.Y. received funding from Albert Einstein College of Medicine to support travel to Sweden for this project.

## Supporting information




**Extended data Figure S1**: Exposure response function for same‐day temperature percentile and OHCA: sensitivity analyses. In these sensitivity analyses, the figures depict the exposure response functions of same‐day temperature percentile and OHCA among cases (a) occurring at home; (b) cases occurring away from the home; (c) adjusting for PM_2.5_; (d) with knots at the 10th, 50th, 75th, and 90th percentiles of temperature; (e) with knots at the 25th, 50th, and 75th percentiles of temperature; and (f) with knots at the 25th, 50th, and 90th percentiles of temperature.
**Extended data Figure S2**: Exposure response function for same‐day temperature percentile and OHCA: seasonal. These analyses examine the exposure response function of same‐day temperature percentile and OHCA (a) in the warm season (April–September); (b) cumulative temperature exposure lag of 0–6 days and OHCA: warm season (April–September); (c) same‐day temperature percentile and OHCA: cold season (October–March); and (d) cumulative temperature exposure lag of 0–6 days and OHCA: cold season (October–March).
**Extended data Figure S3**: Odds ratio of OHCA and selected same‐day temperature percentiles by year. These analyses examined year‐on‐year trends in the relationship between OHCA and extreme cold (a, 1st percentile vs. MMP) and extreme heat (b, 99th percentile vs. MMP). No year‐on‐year strengthening or attenuation of the relationship between extreme temperature and OHCA was observed.

STROBE Statement—Checklist of items that should be included in reports of *case‐control studies*.

## Data Availability

Data from this study are available from the corresponding author on reasonable request and approval by the Swedish Ethical Review Authority.
