# Supplementary material for: Ambient air temperature and out‐of‐hospital cardiac arrest: A national case‐crossover study
Source: J Intern Med. 2026 Aug 3;300(4):489–98. doi: 10.1111/joim.70144 (PMC13569376; doi:10.1111/joim.70144)
Supplement: Supplementary file 2 [file JOIM-300-489-s002.docx]

a b c d e f

Extended Data Fig. 1 Exposure response function, same day temperature percentile and OHCA, sensitivity analyses

In these sensitivity analyses, the figures depict the exposure response functions of same day temperature percentile and OHCA among cases (a.) occurring at home; (b.) cases occurring away from the home; (c.) adjusting for PM_2.5_; (d.) with knots at the 10^th^, 50^th^, 75^th^, and 90^th^ percentiles of temperature; (e.) with knots at the 25^th^, 50^th^, and 75^th^ percentiles of temperature; and (f.) with knots at the 25^th^, 50^th^, and 90^th^ percentiles of temperature.

a b c d

Extended Data Fig. 2 Exposure response function, same day temperature percentile and OHCA, seasonal

These analyses examine the exposure response function of same day temperature percentile and OHCA (a.) in the warm season (April-September); (b.) cumulative temperature exposure lag 0-6 days and OHCA, warm season (April-September); (c.) same day temperature percentile and OHCA, cold season (October-March); and (d.) cumulative temperature exposure lag 0-6 days and OHCA, cold season (October-March).

a

b

Extended Data Fig. 3 Odds ratio of OHCA and selected same day temperature percentiles by year

These analyses examined year on year trends in the relationship between OHCA and extreme cold (a, 1^st^ percentile vs MMP) and extreme heat (b, 99^th^ percentile vs MMP). No year on year strengthening or attenuation of the relationship between extreme temperature and OHCA was observed.
